# Laser Indirect Shock Welding of Fine Wire to Metal Sheet

**DOI:** 10.3390/ma10091070

**Published:** 2017-09-12

**Authors:** Xiao Wang, Tao Huang, Yapeng Luo, Huixia Liu

**Affiliations:** School of Mechanical Engineering, Jiangsu University, Zhenjiang 212013, China; huangtao102510@163.com (T.H.); Yapengluo@126.com (Y.L.); lhx@ujs.edu.cn (H.L.)

**Keywords:** laser indirect shock welding, wire, metal sheet, interface, tensile shear test, nanoindentation hardness

## Abstract

The purpose of this paper is to present an advanced method for welding fine wire to metal sheet, namely laser indirect shock welding (LISW). This process uses silica gel as driver sheet to accelerate the metal sheet toward the wire to obtain metallurgical bonding. A series of experiments were implemented to validate the welding ability of Al sheet/Cu wire and Al sheet/Ag wire. It was found that the use of a driver sheet can maintain high surface quality of the metal sheet. With the increase of laser pulse energy, the bonding area of the sheet/wire increased and the welding interfaces were nearly flat. Energy dispersive spectroscopy (EDS) results show that the intermetallic phases were absent and a short element diffusion layer which would limit the formation of the intermetallic phases emerging at the welding interface. A tensile shear test was used to measure the mechanical strength of the welding joints. The influence of laser pulse energy on the tensile failure modes was investigated, and two failure modes, including interfacial failure and failure through the wire, were observed. The nanoindentation test results indicate that as the distance to the welding interface decreased, the microhardness increased due to the plastic deformation becoming more violent.

## 1. Introduction

With the rapid development of the micro-electromechanical and medical instruments industry, joints are often made between components of considerably different geometries and sizes. A common example is the joining of a fine wire to a metal sheet, such as an electrode slice and connector [1]. Generally, several different welding methods can be applied to this occasion involving resistance microwelding, wire bonding, and laser microwelding. Friis et al. [1] welded 316L stainless steel wire to a block through resistance microwelding and found that current has a significant influence on joint formation, and the softening of materials was induced. Chen [2] studied resistance microwelding of 316L stainless steel to Pt wire and investigated the joint breaking force, fracture mode, and interfacial metallurgical phenomena. Mo et al. [3] focused on the mechanism of resistance microwelding of insulated copper wire to phosphor bronze sheet. They investigated the effects of the main process parameters and joint microstructure. Yoo et al. [4] studied Ag bonding wire with an Al bond pad and characterized the bondability and interface reactions, and two kinds of intermediate phases were observed. Shi et al. [5] conducted experimental research on laser microwelding of a fine copper wire to an Al pin of an integrated circuit chip.

However, for the above welding technologies, the formation of intermetallic compounds and the existence of the heat-affected zone will severely deteriorate the mechanical properties of welding materials in the welding interface region. Additionally, the thermal cycling during the welding process will lead to the softening of the materials due to the resolidification or recrystallisation [1]. Therefore, a reliable bonding process for interconnection between fine wire and metal sheet is highly desirable.

Shock welding technology is based on solid state shock welding, which has been established as reliable, fast, and cost-effective. In contrast to the traditional welding methods, the principle of the shock welding is based on the jetting effect that atomically cleans metal surfaces to weld each other under ultrahigh shock pressure [6,7]. Accordingly, the heat-affected zone and the formation of intermetallic compounds can be greatly reduced, and excellent welding quality can also be obtained for dissimilar material combinations with very different melting points [8,9,10].

Generally, there are two types of shock welding technologies, namely, explosive welding (EXW) and magnetic pulse welding (MPW). Gülenç et al. [11] produced wire-reinforced composite materials through explosive welding in which the wire mesh was used as reinforcement to improve the mechanical properties of the explosively-welded aluminum plates. Zhou et al. [12] investigated the ballistic resistance of steel-wire reinforced two-layer explosively-welded plates and found that the ballistic resistance of that was greatly improved compared with the same thickness target without reinforced steel-wire. Zhang et al. [13] made the lap joint with embedded wires through magnetic pulse welding. The embedded wires were attached to the target plate prior to welding and these were used to make the flyer contact onto the target with a certain impact angle.

Although the above two shock welding processes involve the welding of wire and plate, the wire merely acts as the intermediate layer and these two types of methods are mainly applicable to the form of plate/plate, especially large-sized metal plates [10]. However, the spot welding of wire/sheet is at a very small size. Hence, the above two shock welding processes would not be a good choice for the spot welding of wire/sheet.

**Figure 1:**
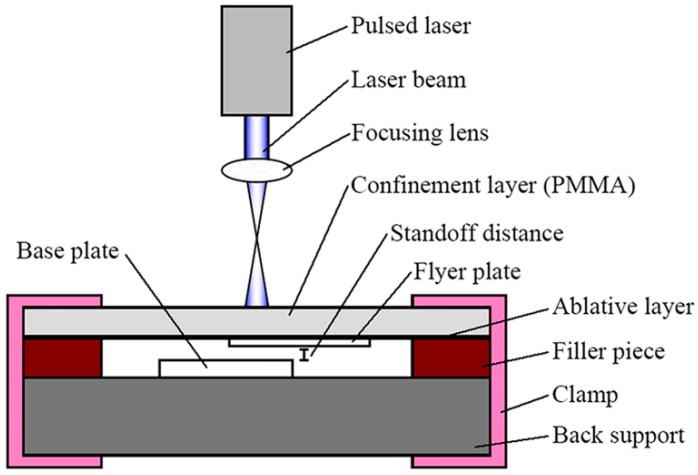
Schematic diagram of laser impact spot welding.

In recent years, laser shock welding (LSW) which is a spot welding technique has been attracting more and more attention. Daehn and Lippold [14] proposed LSW and found that the laser shock spot welding can be applied to relatively thin sheets (about 200 μm or less, and welding regions of a few millimeters in diameter). By means of LSW, Zhang et al. [10] investigated the welding ability of 50 μm thick AA1100 plate and low-carbon steel 1010 plate, and found that the interface was nearly flat for LSW. A varied thickness (25–250 μm) Al flyer was successfully welded with a Ti target by Wang et al. [15]. Afterwards, Wang et al. [16] successfully welded 100 μm thick aluminum plate to 100 μm thick copper plate with the angle welding setup. Wang et al. [17] then developed the laser impact spot welding technique shown in Figure 1, and welded 50 μm thick aluminum plate to 100 μm thick copper plate. From the above, their studies showed that the LSW technique has outstanding advantages in spot welding dissimilar metal sheets with smaller thicknesses at the micrometer scale. This makes the technique promising for applications in the welding of fine wire to metal sheet.

However, the present apparatus of the laser shock welding technique still has some problems: in the welding process shown in Figure 1, the laser beam reacts on the flyer plate directly, which will ablate the surface of the flyer plate, causing poor surface smoothness [17] and destroying the surface quality. In addition, the flyer plate is stuck to the ablative layer with double-sided sticky tape or cyanoacrylate adhesive before welding. Furthermore, a connecting layer will remain on the welding spots after welding and has to be cleared, especially when cyanoacrylate adhesive is used. This will greatly reduce welding efficiency. In order to solve these problems, a protective medium is required to add between the flyer plate and the ablative layer to protect the metal sheet from being ablated and simplify the experimental procedure.

This paper introduces laser indirect shock welding (LISW) of fine wire to metal sheet, in which the metal sheet was propelled by the driver sheet toward the wire to obtain metallurgical bonding under a laser-induced shockwave. This process utilized silica gel as a driver sheet, which was sprayed with black paint before welding and then placed on the metal sheet. Therefore, it used the driver sheet to propel the metal sheet indirectly instead of the direct shock of the laser, thus preventing the metal sheet from being ablated. Al sheet/Cu wire and Al sheet/Ag wire were welded together by laser indirect shock welding (LISW). The morphologies of the welded samples were observed and the welding interface of laser indirect shock welding joint was investigated. In addition, the connection strength of welded samples was tested by tensile shear test. Finally, the nanoindentation test was implemented to study microhardness variation near the welding interface.

## 2. Mechanism of Laser Indirect Shock Welding

**Figure 2:**
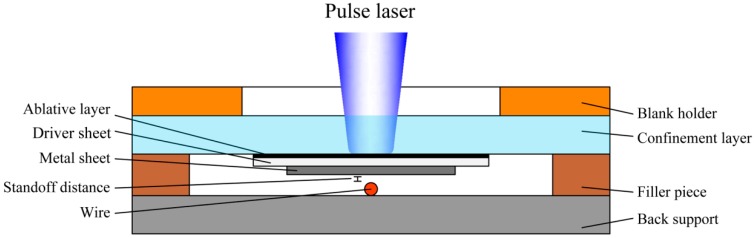
Schematic diagram for laser indirect shock welding.

The basic schematic diagram of laser indirect shock welding of fine wire to metal sheet is shown in Figure 2. The experimental setup mainly consists of a blank holder, confinement layer, ablative layer, driver sheet, metal sheet, wire, back support, and filler piece. When the pulsed laser beam transmits through the transparent confinement layer and focuses on the ablative layer, the irradiated ablative layer is heated and then instantaneously vaporizes into the high-temperature and high-pressure plasma. The resulting plasma confined by the confinement layer expands quickly and changes into laser induced shockwave between the confinement layer and the driver sheet. The shockwave will act on the metal sheet after propagating into the driver sheet and then propel the metal sheet toward the wire in several nanoseconds. In the standoff distance, the metal sheet accelerates downwards and begins to shock the wire. Since the surface of metal sheet is flat and the surface of the wire is a round arc, there will be shock angle at the collision point of the sheet/wire. When the shock angle and the shock velocity increase to a certain value, the jetting is generated which will clean away the surface oxide layer and bring the two fresh surfaces into atomic distance under laser-induced shockwave pressure [17]. Then, solid state bonding is obtained.

In the course of LISW, the confinement layer can prolong the interaction time with shockwave; the ablative layer improves the laser absorptivity and the efficiency of plasma conversion; and the driver sheet acting on the metal sheet converts optical energy of the laser beam into mechanical energy of the shockwave.

## 3. Experimental Preparation and Equipment

### 3.1. Experimental Preparation

Copper wires (diameter: 0.15 mm), silver wires (diameter: 0.15 mm) and 1060 pure aluminum sheets (8 mm × 8 mm × 0.1 mm) were used in the experiment. The chemical compositions of materials are given in Table 1, Table 2 and Table 3. The chemical composition of aluminum sheet is provided by Shanghai Fengqi Metallic Materials Co., Ltd. (Shanghai, China) and the chemical compositions of copper wire and silver wire are provided by Beijing Huanqiu Jin Xin International Technology Co., Ltd. (Beijing, China). All sample materials were cleaned with anhydrous alcohol before welding process. The experimental setup was fixed on a XYZ workbench. The distance between the focusing lens and the ablative layer can be adjusted to control the diameter of laser spot. The laser spot with a diameter of 1.5 mm was used in this experiment. The detailed specimen parameters and experimental conditions are given in Table 4.

To prevent the leakage of plasma, a blank holder with 12 N force was used in the experiment. Due to its high transmittance, K9 glass with the thickness of 6 mm was used as the confinement layer. The silica gel was utilized as driver sheet, the thickness of which was 100 μm. A thin layer of black lacquer was selected as the ablative layer, which was sprayed on the upper surface of the driver sheet before driver sheet’s connection to the confinement layer. Then the upper surface of the driver sheet was pressed on the confinement layer whose surface was wet and the driver sheet was sprayed with black paint so that the confinement layer can stick under the action of atmospheric pressure. Subsequently, the Al sheet was pressed on the lower surface of the driver sheet and the Al sheet was stuck to the lower surface of the driver sheet by van der Waals forces. The wire was fixed on the back support right against the metal sheet with double-sided sticky tape.

### 3.2. Experimental Equipment

**Figure 3:**
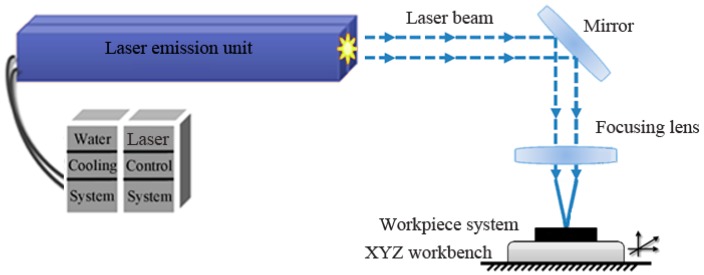
Experimental setup of laser indirect shock welding.

A Spitlight 2000 Nd:YAG laser (InnoLas Corporation, München, Germany) with a Gaussian distribution beam was utilized for the LISW experiments, as shown in Figure 3, and its main parameters are listed in Table 5.

After the LISW process, the samples used for metallographic analysis were fixed by a cold inlaid technique, then the inlaid specimens were mechanically polished using five grades of abrasive papers (JIS #80, #400, #1200, #2000, and #3000) and finished using 0.5 μm particle diamond polishing agent. Cross-sections and longitudinal-sections of the welds were observed using optical microscopy with KEYENCE VHX-1000C microscope (KEYENCE Corporation, Osaka, Japan). The surface morphology and the welding interface of different material joints were investigated using a scanning electron microscope (SEM, Hitachi Corporation, Tokyo, Japan). Additionally, the elemental analysis of the welding interface was examined using an energy dispersive spectroscopy (EDS, EDAX Corporation, Mahwah, NJ, USA).

**Figure 4:**
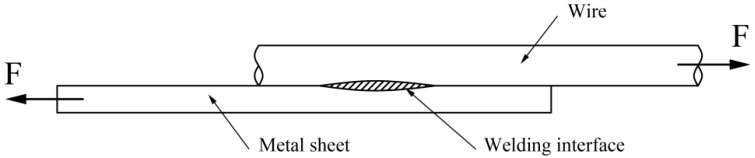
Schematic of tensile shear test.

To examine the mechanical property of the joints, a tensile shear test was performed on an Instron Type UTM 4104 testing machine (SUNS Corporation, Shenzhen, China) with a pull speed of 2 mm/min at room temperature and standard atmospheric pressure. The tensile shear test setup is shown schematically in Figure 4. The displacement and load force were recorded during the tensile shear test.

In order to characterize the microhardness in the welding interface region, nanoindentation hardness test was conducted on a NanoIndenter CSM (Anton Paar, Graz, Austria). The maximum loading force was set as 8 mN, and the maximum load was kept for 10 seconds. The loading and unloading speed were both set as 16 mN/min. The test points were selected every 10 μm in the direction perpendicular to the bonding interface. Three points were tested to obtain the average value of the hardness on every test position.

## 4. Experimental Results and Discussion

### 4.1. Morphology of Welding Examples

**Figure 5:**
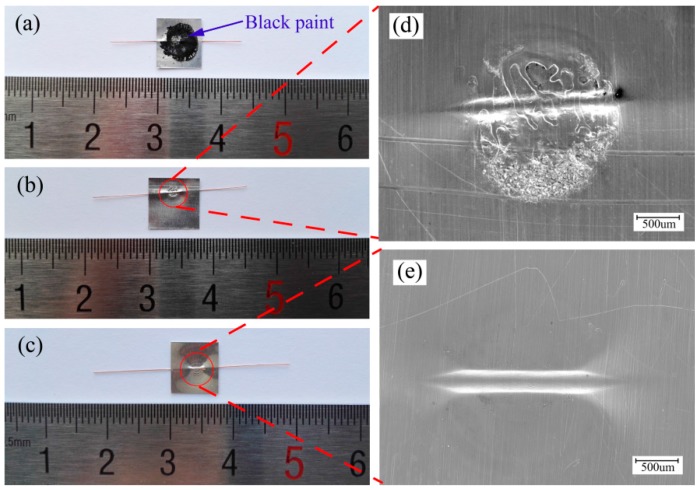
Upper surfaces of welding samples of Al sheet/Cu wire (laser pulse energy: 1380 mJ): (**a**) sample without removing paint under the process in which the laser shocked directly; (**b**,**d**) sample after removing paint under the process in which the laser shocked directly; (**c**,**e**) sample under the process in which the laser shocked indirectly.

Figure 5a,b,d presents upper surfaces of the welding combination of Al sheet/Cu wire using a 1380 mJ laser pulse energy under the process (shown in Figure 1) in which the laser beam shocked the Al sheet directly. As shown in Figure 5a, the surface of the Al sheet was covered with black paint after welding since the Al sheet was stuck to black paint with cyanoacrylate adhesive. Therefore, the welding samples must be cleaned with paint stripper and alcohol. Figure 5b,d presents the welding sample that has been cleaned. Due to the laser shocking the Al sheet directly, the surface of the Al sheet was ablated. Therefore, this is disadvantageous to guarantee the surface quality of the welding samples. 

**Figure 6:**
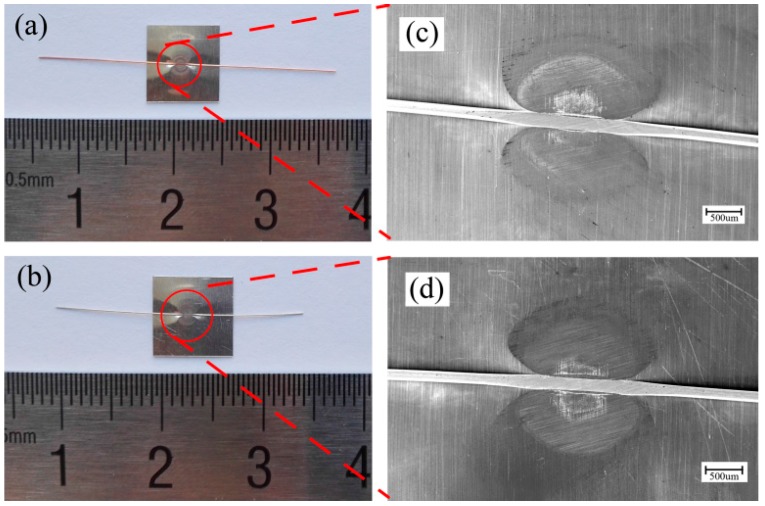
Bottom surfaces of welding samples under the process in which the laser shocked indirectly (laser pulse energy: 1380 mJ): (**a**,**c**) Al sheet/Cu wire; (**b**,**d**) Al sheet/Ag wire.

For explosive welding [6], the flyer plate was covered by a buffer which was a thin sheet of rubber and then the buffer can protect the surface of the flyer plate free from direct action of the explosive. Therefore, the use of a driver sheet may be able to solve the above problems and it can make the laser shock the Al sheet indirectly, as shown in Figure 2. The employment of a driver sheet can play the role of the protective medium, which ensures the surface quality of Al sheet. Figure 5c,e presents upper surfaces of the welding combination of Al sheet/Cu wire, in which the driver sheet was used in the welding process and the rest of experimental conditions were identical with that in the process in which the laser shocked the Al sheet directly. As can be seen, the laser shock region of Al sheet maintained high surface quality and smoothness. Figure 6 presents bottom surfaces of the welding combinations of Al sheet/Cu wire and Al sheet/Ag wire using 1380 mJ laser pulse energy. As can be seen, the adopted system and method can successfully weld a fine copper/silver wire onto an aluminum sheet under the process of LISW.

### 4.2. Welding Interface

**Figure 7:**
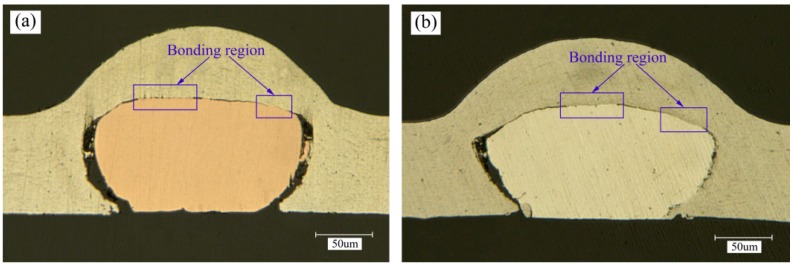
Optical micrographs of the welding cross-section (laser pulse energy: 1020 mJ): (**a**) Al sheet/Cu wire; (**b**) Al sheet/Ag wire.

**Figure 8:**
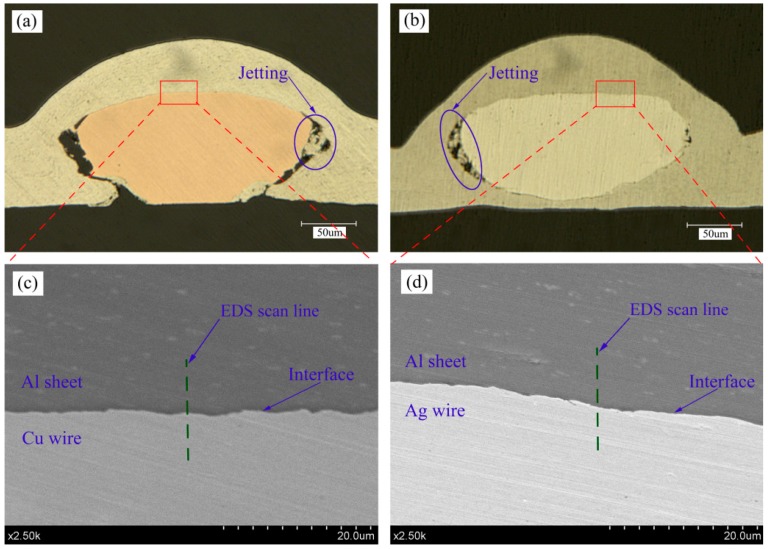
Optical micrographs of the welding cross-section (laser pulse energy: 1550 mJ): (**a**) Al sheet/Cu wire; (**b**) Al sheet/Ag wire. SEM images of the welding cross-section at the interface (laser pulse energy: 1550 mJ): (**c**) Al sheet/Cu wire; and (**d**) Al sheet/Ag wire.

The optical micrographs of the cross-section of two different welding combinations under different laser pulse energies are shown in Figure 7 and Figure 8. As shown in Figure 7, when the laser pulse energy was 1020 mJ, the bonding region was only in some small areas and discontinuous. When the laser pulse energy was increased to 1550 mJ, the bonding region comprised the whole shock area between the wire and the metal sheet and the bonding region was increased, as shown in Figure 8a,b. The reason why increasing laser pulse energy can increase the bonding zone can be explained as follows: due to the increase of laser pulse energy, the maximum shock velocity increases, thereby increasing the scope of the effective shock velocity. A similar conclusion in laser impact spot welding of sheet/sheet was reported by Liu et al. [18].

It is interesting that the bottom of the Ag wire can also be welded to the Al sheet, however, this cannot be achieved in the welding combination of Al sheet/Cu wire under the same laser pulse energy, as shown in Figure 8a,b. The reason for this phenomenon can be expressed as follows: the plasticity of the Ag wire is better than that of the Cu wire, therefore, the Ag wire is easier to deform. As shown in Figure 8a,b, the Ag wire is squashed more seriously than the Cu wire after shock welding and this is convenient for more Al material to squeeze into the bottom of the Ag wire, thus, shock welding can be accomplished between the bottom of Ag wire and the Al material under the shockwave pressure. It is an advantage that the bottom of Ag wire can also be welded to the Al sheet, which can greatly increase the bonding area between them. For example, in electrical connections in small-scale electronic devices, the larger the contact area is, the smaller the electric resistance will be [19].

On the side of the wires, the jetting was observed as shown in Figure 8a,b. When the Al sheet impacted the wire, a high-speed jetting required to remove the surface oxide was formed at the collision point. Then, two fresh surfaces were formed and brought into atomic distance under shock pressure. Accordingly, metallurgical bonding can be obtained. During the shock welding course, the jetting flowed outward and eventually stopped in the gap between the wire and the Al sheet. Most researchers found that in order to achieve successful welding, the jetting must be generated in the welding process [20,21,22]. Therefore, the jetting is essential for the shock welding between wire and metal sheet.

SEM micrographs in Figure 8c,d display the welding interfaces of two different welding combinations in Figure 8a,b, respectively. The interfaces are nearly flat and the waves are small. Generally, there are three kinds of interface morphologies for shock welding: flat interface, wavy interface and vortex wave interface. According to the study from Grignon et al. [23], for a combination of welding materials, the interface morphology is critically dependent on the process parameters, such as shock velocity and shock angle. Compared with the large energy of explosive welding and magnetic pulse welding, the lower energy of LISW results in lower shock velocity, which is not enough to form the wavy interfaces. Research from Zhang et al. [10] confirmed this finding. In addition, compared with the collision zone of sheet/sheet, the collision zone of the sheet/wire is too narrow to form the wavy interface.

**Figure 9:**
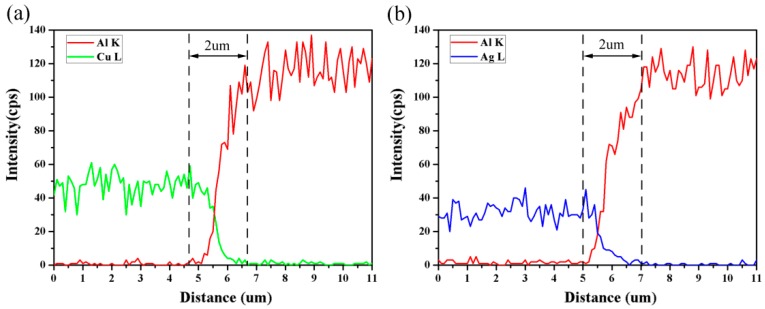
EDS line scan at the interface (laser pulse energy: 1550 mJ): (**a**) Al sheet/Cu wire; (**b**) Al sheet/Ag wire.

The element distribution across the interface was studied by EDS line scan as shown in Figure 8c,d. The EDS results are shown in Figure 9 in which the ‘Cu L’, ‘Ag L’ and ‘Al K’ represent the L line of copper and silver and the K line of aluminum, respectively. At the welding interfaces of Al sheet/Cu wire and Al sheet/Ag wire, the element distribution exhibits a gradual transition(X shape) rather than changing from one phase to other phase with several steps, which can illustrate that the intermetallic phases are absent. No intermetallic phases generating at the welding interface can guarantee the welding strength of the interface because the intermetallic phases can produce brittle damage [10]. In addition, a short element diffusion layer with width of approximately 2 μm emerges at the sheet/wire welding interface during shock welding, as shown in Figure 9. A short element diffusion layer was also detected by Wang et al. [24]. There are some advantages for element diffusion: on the one hand, element diffusion is advantageous to narrow the distance between two metal atoms and then keep a balance between attraction and repulsion. On the other hand, element diffusion can promote the metallurgical process at the interface. Furthermore, the short diffusion layer also limits the formation of intermetallic phases [25].

**Figure 10:**
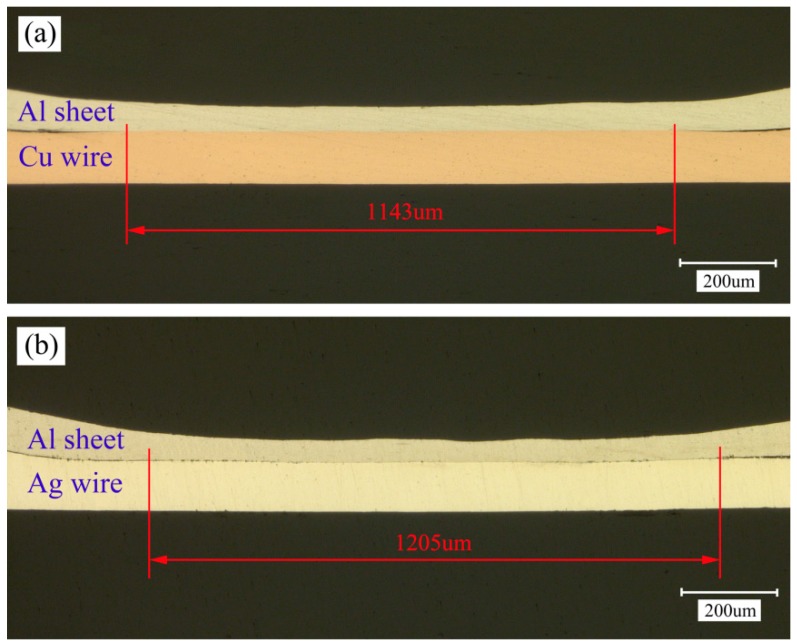
Optical micrographs of the welding longitudinal section (laser pulse energy: 1200 mJ): (**a**) Al sheet/Cu wire; (**b**) Al sheet/Ag wire.

Figure 10 shows the longitudinal section of different welding combinations under 1200 mJ laser pulse energy. It can be observed that the welding lengths of the Al sheet/Cu wire and the Al sheet/Ag wire were 1143 μm and 1205 μm, respectively. The welding lengths were a bit shorter than the laser spot diameter (1500 μm). Due to the Gaussian distribution of the laser beam, the edge of the metal sheet is irradiated with lower laser pulse energy, which is not enough to accelerate the edge region of the sheet to effective shock velocity.

### 4.3. Tensile Shear Test

**Figure 11:**
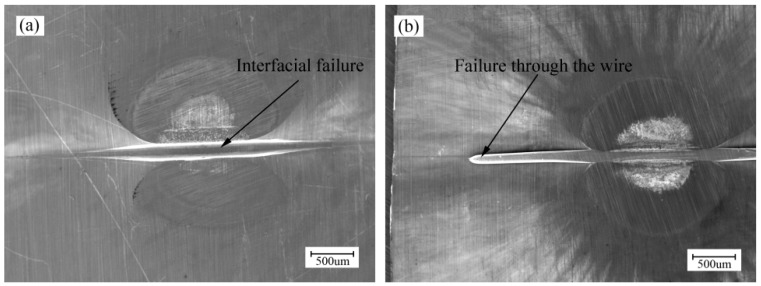
Failure modes of the welding samples under different laser pulse energies: (**a**) interfacial failure (1020 mJ); (**b**) failure through the wire (1550 mJ).

**Figure 12:**
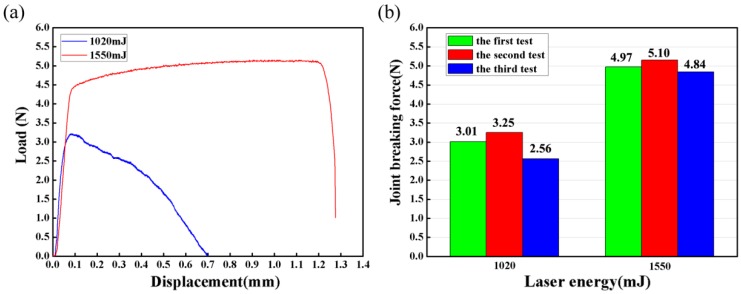
(**a**) Load-displacement curves measured from single tensile shear test of Al sheet/Cu wire; (**b**) joint breaking force of Al sheet/Cu wire under different laser pulse energies.

There were two failure modes in the tensile shear tests of Al sheet/Cu wire, as seen in Figure 11. Figure 11a shows the interfacial failure and Figure 11b shows the failure through the wire. Interfacial failure indicates the low strength of the joint, while failure through the wire indicates adequately strong bonds. It can be illustrated by measuring load-displacement curves for interfacial failure using 1020 mJ laser pulse energy and failure through the wire using 1550 mJ laser pulse energy, as shown in Figure 12a. The sudden decrease in tensile force indicated the sheet/wire interface fracture. The wire fracture showed a typical ductile fracture mode with slow decrease in tensile force and the maximum load force was about 5.1 N, which was much larger than that of the interface fracture.

When the laser pulse energy was 1020 mJ, the bonding region was only some small parts as shown in Figure 7a, which resulted in the weak bond of Al sheet/Cu wire. Thus, interfacial failure is more likely to occur at the low laser pulse energy. When the laser pulse energy was increased to 1550 mJ, the bonding region was the whole shock area between the wire and the metal sheet and the bonding region was increased, as shown in Figure 8a, which resulted in the strong bond of the Al sheet/Cu wire. Hence, failure through the wire may take place at the high laser pulse energy. The failure mode changes from interfacial failure to failure through the wire due to the increase of the effective welding area. Another reason is that the increase of laser pulse energy may result in the increase of microhardness near the welding interface, thereby increasing the strength and then affecting the failure mode.

The tensile shear tests were carried out three times for two different laser pulse energies. Figure 12b shows the joint breaking force of all the performed tests under 1020 mJ laser pulse energy and 1550 mJ laser pulse energy. The average failing load under 1020 mJ laser pulse energy was 2.94 N and that under 1550 mJ was 4.97 N.

Additionally, the same failure characteristics were also observed for the welding combination of Al sheet/Ag wire.

### 4.4. Microhardness Variation

**Figure 13:**
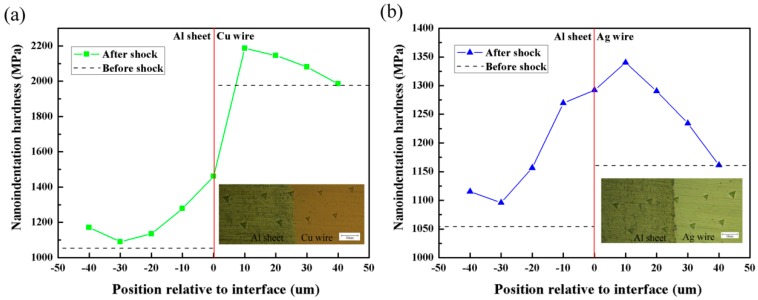
Nanoindentation hardness profile near the welding interface (laser pulse energy: 1550 mJ): (**a**) Al sheet/Cu wire; (**b**) Al sheet/Ag wire.

The nanoindentation hardness of Al sheet, Cu wire and Ag wire (raw materials) are 1055.4 MPa, 1985.5 MPa, and 1161.3 MPa, respectively. The nanoindentation hardness values measured in the welding interface region are illustrated in Figure 13. It can be seen that both in the welding combinations of Al sheet/Cu wire and Al sheet/Ag wire under 1550 mJ laser pulse energy, the nanoidentation hardness of the test positions is clearly greater than the raw materials. As the distance to the welding interface decreases, the nanoidentation hardness increases gradually. Due to the high speed collision process of LISW, there is heavy plastic deformation occurring near the welding interface. As the distance to the welding interface decreases, the degree of plastic deformation will increase, which contributes to the corresponding degree of improvement of the nanoidentation hardness.

As seen in Figure 13a, the nanoidentation hardness of Al sheet/Cu wire at −10 μm and 10 μm position increase 21.1% and 10.1%, respectively, compared to the raw materials. From Figure 13b, the microhardness variation of Al sheet/Ag wire is similar to that of Al sheet/Cu wire. The nanoidentation hardness of Al sheet/Ag wire at −10 μm and 10 μm position increase 20.3% and 15.4%, respectively. Moreover, the sudden shock of laser can also contribute to the increase of microhardness on the surface of Al sheet. As can be seen from Figure 13, the nanoidentation hardness on the surface of Al sheet (position −40 μm) is increased obviously. This phenomenon is similar to the sudden shock of explosion causing the hardness increase in the upper face of the flyer plate in explosive welding [26].

There are some other reasons for the increase of microhardness near the welding interface: on the one hand, the bonding area may experience quick cooling during the welding process, which can be considered as a quenching course [16]. On the other hand, due to the ultrahigh strain rate process of LISW, the microstructure evolution such as dislocation, twin boundary, and grain refinement of the materials may occur near the welding interface [27]. On the contrary, for resistance microwelding of wire to sheet, the hardness near the welding interface is fairly smaller compared with the base metal, because the thermal cycling during welding process leads to the softening of the materials due to the resolidification or recrystallisation [1]. Moreover, the formation of intermetallic compounds and the existence of the heated affected zone will decrease the hardness near the welding interface. Therefore, this is also a great superiority for LISW compared with laser microwelding and resistance microwelding in the welding of fine wire to metal sheet.

## 5. Conclusions

In this paper, a feasible welding technique of fine wire to metal sheet was presented, which employed the driver sheet to accelerate the metal sheet toward the wire to get metallurgical bonding under the laser induced shockwave. The feasibility of this novel technology was verified by welding the Cu wire and the Ag wire to the Al sheet with a low laser pulse energy system. In the experiment, driver sheet was utilized to get better results for the welding joints. The welding interface of laser indirect shock welding joint was investigated. The strengths for welding samples were tested by tensile shear test. In addition, the nanoindentation test was implemented to measure the microhardness near the welding interface. The main results are summarized as follows:

(1) Cu wire and Ag wire were successfully welded to Al sheets by LISW. High surface quality was obtained with the use of driver sheet in comparison to focusing the laser beam directly on Al sheet.
(2) With the increase of laser pulse energy, the bonding area of sheet/wire increased. The bottom of the Ag wire can also be welded to the Al sheet due to the good plasticity of the Ag wire. Jetting was observed and was essential for the shock welding between wire and metal sheet. The welding interfaces are nearly flat and the waves are small. According to the EDS analysis, the intermetallic phases are absent and a short element diffusion layer emerges at the sheet/wire welding interface.
(3) There were two failure modes in the tensile shear tests. Samples welded by 1020 mJ failed through the interface and samples welded by 1550 mJ failed through the wire. According to the load-displacement curves, failure through the wire indicates adequately stronger bonds than failure through the interface.
(4) As the distance to the welding interface decreases, the microhardness increases gradually. The microhardness measured near the interface was obviously increased owing to the heavy plastic deformation, cold quenching, and microstructure evolution. In addition, the sudden shock of the laser can also contribute to the increase of microhardness on the surface of Al sheet.

## Figures and Tables

**Table 1:** Chemical composition of aluminum sheet (wt %).

Al	Mn	Si	Cu	Mg	Fe
99.2	0.05	0.25	0.05	0.05	0.4

**Table 2:** Chemical composition of copper wire (wt %).

Cu	Bi	Sb	As	Fe	Pb	S	Other
99.9	0.001	0.002	0.002	0.005	0.005	0.005	0.01

**Table 3:** Chemical composition of silver wire (wt %).

Ag	Cu	Bi	Fe	Pb	Sb	Pd	Se	Te
99.99	0.003	0.0008	0.001	0.001	0.001	0.001	0.0005	0.0005

**Table 4:** Detailed specimen parameters and experimental conditions.

Parameters	Values
Material combinations	Al sheet/Cu wire, Al sheet/Ag wire
Sheet size (mm)	8 × 8 × 0.1
Diameter of wire (mm)	0.15
Standoff distance (mm)	0.2
Diameter of laser spot (mm)	1.5
Laser pulse energy (mJ)	1020, 1200, 1380, 1550

**Table 5:** Main parameters of Spitlight 2000 Nd:YAG laser.

Parameters	Values
Pulse energy	80–1800 mJ
Pulse Width	8 ns
Wave Length	1064 nm
Exit spot diameter	9 mm
Energy Stability	<±1%
